# Fair Topologies: Community Structures and Network Hubs Drive Emergence of Fairness Norms

**DOI:** 10.1038/s41598-017-01876-0

**Published:** 2017-06-02

**Authors:** Mohsen Mosleh, Babak Heydari

**Affiliations:** School of Systems and Enterprises, Stevens Institute of Technology, Hoboken, NJ 07030 USA

## Abstract

Fairness has long been argued to govern human behavior in a wide range of social, economic, and organizational activities. The sense of fairness, although universal, varies across different societies. In this study, using a computational model, we test the hypothesis that the topology of social interaction can causally explain some of the cross-societal variations in fairness norms. We show that two network parameters, namely, community structure, as measured by the modularity index, and network *hubiness*, represented by the skewness of degree distribution, have the most significant impact on emergence of collective fair behavior. These two parameters can explain much of the variations in fairness norms across societies and can also be linked to hypotheses suggested by earlier empirical studies in social and organizational sciences. We devised a multi-layered model that combines local agent interactions with social learning, thus enables both strategic behavior as well as diffusion of successful strategies. By applying multivariate statistics on the results, we obtain the relation between network structural features and the collective fair behavior.

## Introduction

Human’s deep presuppositions of fairness and inequality are proven in a wide range of contexts, such as the relationship between relative income and reported job satisfaction^1^, happiness^2^, health and longevity^3^, and reward-related brain activity^4^. These attitudes towards fairness play an important role in governing human behavior in social, economic, and organizational activities^5–7^. Earlier studies on fairness norms that were largely restricted to industrial societies showed relatively small fairness variation among different populations, suggesting that fairness norms were mostly the result of universal behavior patterns. This was later challenged by the seminal work of Henrich *et al*.^8^ and a number of other studies that show significant variations in fairness attitudes across different societies^9, 10^ and organizations^11–13^. These studies hypothesize the role of certain social and institutional factors on the scale and intensity of fairness norms^8, 14–16^. However, a systematic explanation of variation in fairness norms across different human societies has remained an open question that motivates our paper. Here, we hypothesize that the overall structure of social interactions, as captured by the network topology, can causally influence fairness norms and can also be linked to the hypothesized factors that have been suggested by earlier empirical studies. We take a computational approach to investigate the role of macro-level structural features of social networks in explaining the inter-societal variation of fairness norms.

The variations of fairness across societies have been largely attributed to cultural differences, yet interpretations have been mixed^16–18^. Cross-country results of experimental studies using canonical economic games under different conditions of the balance of power between two parties suggest that difference in the national culture results in differing beliefs about fairness^19^. Studies that attempted to attribute variations of these beliefs to country-level *cultural variables* ^20, 21^ show a relationship between the behavior of individuals and *the scale of respect for authority* ^15^. Moreover, recent experimental studies tentatively suggest religion as one of the influential factors affecting individuals’ fair behavior^22^. As a canonical paradigm to investigate fairness, the majority of these studies use the Ultimatum Game.

The Ultimatum Game (UG hereafter) has been the bedrock of a large number of studies that investigate the systematic deviation of human altruistic behavior from theoretical models that are based on the *homo economicus* assumption^6, 23, 24^. In the UG, two players are supposed to decide about sharing a fixed amount of money; one player acts as the *Proposer* and the other plays the role of the *Responder*. The proposer offers a split of the amount and the responder accepts or rejects. No bargaining is allowed. If the responder accepts, both players receive their amount based on the split; otherwise neither player receives anything. A rational responder, seeking to maximize her utility, would accept even the smallest positive offer. Being aware of the rationality of his partner, a rational proposer would therefore claim almost the entire amount. However, the results of a large number of experimental studies using the UG contradict with the prediction of pure rational models; most proposers offer a fair share and most responders reject low (but nonzero) offers^25–27^. The division of the money in the UG represents a measure for fairness norms.

Experimental studies based on the Ultimatum Game have reported significant cross-societal variations in average size of the offers by the proposers and the rejection rates by the responders. Table 1 shows the results of a number of key empirical studies of variation of fairness norms across different societies together with attributed institutional factors. The variation has particularly been bolder when experiments are conducted in non-industrial societies. In^8^ for example, the UG was played in 15 small-scale societies; the mean offer varied between 0.26 to 0.58 (as a proportion) and the rejection rate for low offers (offers of 0.2 or less) had a range between 0 to 0.8. Later studies conducted in other non-industrial societies have shown comparable large variations^14^. What social and institutional factors cause such variations? One potential key determinant is the macro-level structure of social networks. Experimental studies have previously shown that network structure of social interaction has a significant impact on evolution of collective behavior related to cooperation and competition^28^, inequality^29^, and exploration/exploitation^30^. Moreover, empirical studies based on experimental UG suggest factors that can be related to social structure. A series of studies that explored the motivation for fairness in anonymous interactions across dramatically diverse population show that fairness co-varies with *Payoffs to Cooperation* (PC) and *Market Integration* (MI) where PC represents the size of a group’s payoff from cooperation in economic production, and MI represents how much people rely on market exchange in their daily lives as opposed to rely on home-grown or hunted^8^.

**Table 1:** Empirical studies of variation of fairness norms using the Ultimatum Game and attributed social/institutional factors.

Empirical study	Mean offers	Rejection rates	Attributed institutional factors (as mentioned in each study)
15 small-scale societies^8^	0.26–0.58	0–0.8^(2)^	*Payoffs to Cooperation*, *Market Integration*
Multi-village Tanzanian ethnic groups^14^	0.15–0.61	0–0.4^(3)^	*Hierarchy of Chiefdom System*, *Scale of Cooperative Units*
Meta-Analysis of 75 results of UG experiments world-wide^15^	0.26–0.58	0–0.4	Cultural difference (*Respect for Authority*)
Cross-national UG experiment (UK and Malaysia)^16^	0.42–0.46	0.07–0.20^(1)^	Cultural difference (no specific factor is identified)

Mean offers are reported for different treatments and social groups. Offer values are scaled between 0 and 1. (1) rejections as a proportion of all responses. (2) Rejection rates for offers of 0.2 or less. (3) Rejection rates for offers of 0.1 (i.e., 100 Tanzanian shillings).

To explain network topology parameters that affect fairness norms and their cross-society variations, we devised a multi-layered computational model that combines local agent interactions with social learning, thus enables both strategic behavior as well as diffusion of successful strategies. At the heart of this model, agents interact according to a one-shot UG, and update their strategies based on an evolutionary process. The behavior of agents is simulated on a wide range of carefully selected networks that generate variations of structural features and multivariate statistics is used to identify the relation between average strategies of agents at the equilibrium and the network’s structural features.

Our model offers two main contributions: (1) It indicates a causal relationship between changes of structural characteristics of social network and variation of fairness norms. (2) It identifies structural features that are aligned with the findings of empirical studies of inter-societal variations of fairness norms and sheds some light on role of social structure as one of key determinants in explaining the variation of fairness across societies. Concurring with previous computational studies^31, 32^, the results of our study show that structures that are close to all-inclusive interactions (full-graph) exhibit less fairness. Our study, however, identifies two other structural drivers that have been missing in the literature: community structure, represented by network modularity index, and network *hubiness*, represented by skewness of degree distribution of the network. The latter two network parameters also help to further clarify some of the empirical findings that relate social institution parameters to fairness norms.

## Results

To study the effect of structure on agents’ strategies playing the UG, we developed a computational agent-based model based on evolutionary game theory on networks^33^. Each agent has a strategy vector containing the offer value and the acceptance threshold, each varies between 0 and 1; where higher values represent stronger fairness norms. In each evolutionary round, agents interact based on a pairwise UG while using the same strategy vector against all partners and collect payoffs (Equation 1). Once all agents have completed one round of the UG with all their partners, game scores are calculated (Equation 2).

An evolutionary process based on the agents’ game scores (Equation 3) represents learning and strategy adoption by agents. That is, agents stochastically adopt the strategy of their neighbors with higher game scores where the probability of adoption is an increasing function of the difference between the two agents’ scores. The evolutionary process represents cultural evolution through social learning but it can also represent genetic evolution, both of which have been linked to the emergence of fairness in the UG^34^. In the context of cultural evolution, an individual tends to imitate the strategy of another individual with a higher payoff and mutation denotes either exploring a new strategy or incorrectly imitating the strategies used by other individuals. In the context of genetic evolution, individuals are born and die, and mutation creates variation into the gene pool.

The interaction structure of agents is determined by the topology of the network where agents are represented as nodes and only interact with their immediate neighbors in the network. To make simulations computationally efficient, we sparsed the search space of network structures based on key structural features that represent characteristics of social structure (see Supplementary Information for details). The structural features that we selected for this study are network average degree, girth and average shortest path that represent compactness of the society, and transitivity that represents dyadic closures in the population. We use the network modularity index to measure the strength of community structure. Modularity is an emergent structural feature of many evolving networks^35^ and has also been found to have a significant impact on the emergence of prosocial behavior^36, 37^. To capture the role of hierarchy and relative power structure, we measure the degree of *hubiness* of the network calculated based on the degree distribution. Hubiness is a structural feature of a wide range of real networks, such as co-authorship networks^38^, the World Wide Web^39^, and the dependency network of natural languages ^40, 41^, that are formed through the mechanisms of growth and preferential attachment where new nodes prefer to link to the more connected nodes^42, 43^. Huby structures are argued to result in economic efficiency for a certain range of network parameters^44^. In such network structures, the higher tendency to link to highly connected nodes results in higher skewness of degree distribution since several nodes are directly connected to a few central nodes (*hubs*). By changing the parameters of network models including Barabási-Albert Scale-Free^39^, Watts-Strogatz Small-World^45^, Erdős-Rényi Random graph^46^, Tree, and Lattice, we generated 26 network structures that sufficiently cover variations of the structural features (Methods).

In each simulation run, agents’ strategies (i.e., offer value and acceptance threshold) are initialized from a uniform distribution. Agents continue interactions in the network environment (i.e., play the UG and update strategies) until their strategies stabilize for a consecutive window of time. For each network structure, we averaged over strategies at the equilibrium for 1024 initializations, for which average strategy remains robust (see Supplementary Information Section 3). The simulation took approximately 40 minutes for all network structures on a 256-core cluster. The results of the simulation were aggregated in a data table, in which rows represent network structures and columns represent structural features as well as the average values of strategies at the equilibrium (see Supplementary Information Section 4).

In order to find the key structural features that drive fairness in the population of agents, we used Principal Component Analysis (PCA) on the simulation data^37^. PCA helps us to represent the variations of structural features and strategy vectors based on fewer number of linearly uncorrelated components and determine variations of which variables are related. The variables in the PCA include network characteristics (i.e., average degree, average path length, transitivity, modularity, and hubiness) and average strategy vector (i.e., $\tilde{p}$: average offer and $\tilde{q}$: average acceptance threshold) of all runs for a network at the equilibrium. We used modularity index, a scalar value that represents the number of links within communities compared to links between communities^47^, as a measure for community structure. We used the heuristic method in^48^ for calculating the modularity of network structures. To measure *hubiness* of the structures, represented by the lack of symmetry in the degree distribution, we used the coefficient of skewness from^49^ (Methods).

**Figure 1:**
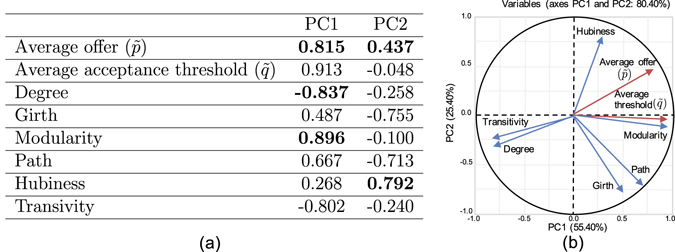
(**a**) Correlations between the initial variables and the independent principal components. Significant correlations are in bold. Average offer is highly correlated with PC1 and moderately correlated with PC2. Modularity is highly correlated with PC1, while Degree is negatively correlated with PC1. Hubiness is highly correlated with PC2 (**b**) Principal Component Analysis results summary. The biplot shows the correlation between initial variables based on the first two principal components. The angles between the variables represent the level correlation. Strategies are initialized from uniform distribution *U*(0, 1) and normalized accumulated payoff per the agent’s degree is used as the game score.

Figure 1 shows the results of the PCA on the correlation matrix constructed from the simulation data. We used the first two components that cumulatively account for 80% of the total variance in the whole data set. Figure 1b shows the *biplot* for the first two components and their correlations with network characteristics and average strategies at the equilibrium. In the biplot, average offer has an opposite direction to the average degree and is approximately orthogonal to the average path length and girth. However, the average offer value is more aligned with modularity and hubiness, suggesting a significant effect of these two structural features. The correlations between network structural features together with average strategies and principal components are provided in Fig. 1a. Average offer is significantly loaded on PC1 and PC2 while modularity is only highly loaded on PC1 and hubiness is highly loaded on PC2. Average degree is negatively loaded on PC1.

**Figure 2:**
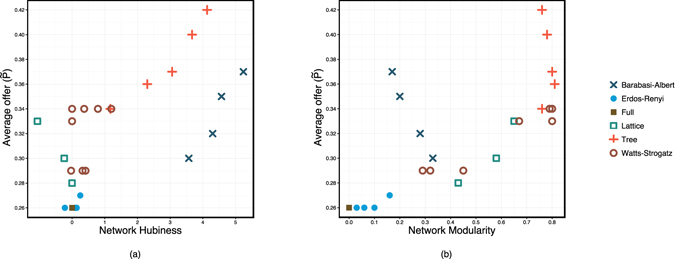
Fairness versus network topology parameters. (**a**) Average offer value vs. network hubiness. (**b**) Average offer value vs. network modularity.

Panel (a) in Fig. 2 shows the level of fairness, measured by average offer value, versus skewness of degree distribution (hubiness). The lowest level of fairness belongs to the Full and Erdös-Rényi networks, and the highest is attributed to Tree structures. The results show that an increase in hubiness led to higher fairness. Variation of hubiness is mainly generated by varying the power of preferential attachment in the Barabási-Albert scale free and the number of children in Tree networks (Supplementary Information Section 1). However, average offers are higher in Tree structures than those in the Barabási-Albert networks. Not only do tree structures have skewed degree distribution but they also have stronger community structure. The rest of the networks with lower hubiness are located along the y-axis; however, the level of fairness varies among these structures. This indicates that hubiness is not the only structural feature affecting the level of fairness. Panel (b) in Fig. 2 depicts the level of fairness versus modularity index and shows that an increase in network modularity results in an increase in the level of fairness. Variation of modularity index has been generated by different lattice structures and Watts-Strogatz network model, in which modularity index varies by changing the probability of rewiring (Supplementary Information Section 1). These structures have similar hubiness and are located along the y-axis in Panel (a). The difference of modularity index and the level of fairness between Tree structures and the Barabási-Albert networks are evident in Panel (b) i.e., Barabási-Albert and Tree structures do not have much variation in modularity index, yet Tree structures have stronger community and exhibit higher level of fairness.

**Figure 3:**
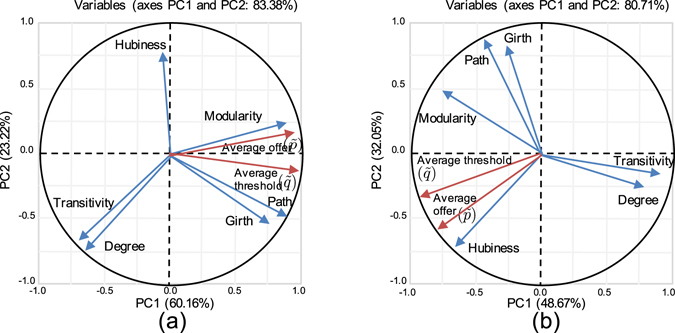
Effects of the initialization and the game score. (**a**) Results of PCA on simulation data when agents’ strategies are initialized from uniform distribution U(0, 0.5), (**b**) results of PCA when total accumulated payoff is used the game score (without normalizing on the agent’s degree).

To find out how the role of structure in evolution of fairness can be affected by other factors, we investigated two other conditions. (1) Since it is less likely that individuals offer/request more than half of the pie, we eliminated large values for initial strategies and used uniform distribution *U*(0, 0.5) instead of *U*(0, 1) for initialization. Figure 3a shows that the effect of hubiness has been eliminated and the average offer value is completely aligned with modularity as a result of initializing the strategies from *U*(0, 0.5). (2) We considered the case where agents do not have information about their partners’ degree and used accumulated payoff (without normalizing by the agents’ degree) in the strategy adoption process. Figure 3b shows that the effect of hubiness is amplified and average offer value is highly correlated with hubiness when accumulated payoff is used as the game score. Under this condition, our simulation results show higher fairness for all network structures used in this study comparing to the case where normalized accumulated payoff by degree is used as the game score.

## Discussion

Our results identify three key structural features of social networks to explain the variation of fairness across different populations: average degree, community structure, and hubiness. Average degree of a network with a fixed number of nodes represents the density of interactions among a population. The results of our model show that populations with a large number of social interactions per individual exhibit lower levels of fairness. This is consistent with previous findings^31, 32, 50^ and is due to the fact that the increase in the density of social interactions leads to a well-mixed population and weakens the effect of structure, thus reduces overall level of fairness.

The intuition behind the role of community structure, captured by network modularity, in variations of fairness is as follows. When the interaction between agents follows a network structure, the success of a strategy *S*_1_ invading a population with strategy *S*_2_ not only depends on the payoff that is obtained from the encounter with the vast majority (i.e., *E*{*S*_2_, *S*_1_} and *E*{*S*_1_, *S*_2_}) but also on local interactions of the invaders (i.e., *E*{*S*_2_, *S*_2_} and *E*{*S*_1_, *S*_2_})^31^. When initial strategies are randomly distributed, a subset of agents will have a fair strategy. A strong community structure would help formation of clusters around agents with similar fair strategies and increases the chance of the fair strategy to take over the population. This concurs with an earlier study that investigates the level of fairness in a range of networks sorted according to a measure of *order* that shows that the level of fairness decreases as the community structure weakens^51^.

Another structural feature that promote fairness in our model is *hubiness*, which is measured by the skewness of degree distribution. Prior studies have shown the significant role of huby structures (structures dynamically generated through the mechanisms of growth and preferential attachment) in the evolutionary dynamics of formation of social norms^52–54^. Our results suggest societies in which few individuals play the role of hubs, which are connected to large number of low-degree individuals, exhibit higher levels of fair behavior in the UG. The intuition is similar to how average degree affects fairness, as explained earlier. Given that higher number of interactions decreases fairness (as the structure becomes closer to a well-mixed population), for a fixed average degree, the structure in which degree of a larger number of nodes is smaller exhibit a higher fairness. That is, the connections are shifted from a majority of nodes to a few hubs while total average degree is constant which results in skewed degree distribution.

Additionally, our model shows that the magnitude of impact for structural drivers of fairness is in general a function of agents’ initial strategies and the information available to individual agents. Our model shows that the rule of hubiness can be degraded when initial values of offers and acceptance thresholds are not larger than half of the pie. We also show that agents’ lack of information about the number of social ties of their neighbors can increase the impact of hubiness. The higher impact of hubiness under this condition is due to the intensified role of hubs in promoting fair strategies. In the evolutionary process of strategy adoption, the strategy of hubs is more likely to be copied by other agents since hubs collect higher game scores due to having large number of interactions (without considering the agent degree in the game score). Since a subset of hubs are initialized randomly to play fair, their strategies are copied even more aggressively by their neighbors, compared to the case when game score is normalized by degree. Further studies can incorporate various conditions of information uncertainty, such as neighbors’ payoffs and neighbors’ local structure, as well as the level of information access (global vs. local) into the model. This can provide insights about how the level of information access in a social network can affect collective fair behavior.

Empirical data that directly supports the effect of social network structure on fairness is often difficult to gather. On the one hand, isolating the effect of social structure from other factors in field experiments is often hard. On the other hand, lab experiments that simultaneously accommodate sufficiently large number of subjects on various network structures is challenging. Despite these limitations, we can attribute some of the factors that are empirically hypothesized to explain fairness in cross-country and cross-organization to social network parameters. Here, we first compare our findings to the results of two empirical studies that demonstrate significant cross societal differences in playing the UG. The first one is a series of papers^8, 22^ led by Henrich based on playing a number of fairness related games, including the UG, in 15 diverse small-scale societies that suggest that societies in which the size of a group’s payoff from cooperation in economic production (*Payoffs to Cooperation*) is higher, and those in which people rely on the market for their daily life (*Market Integration*) have higher levels of fairness. The second study^14^ is based on results of playing the UG in two multi-village Tanzanian ethnic groups (Pimbwe and Sukuma) which are embedded in similar environments and political systems, yet have drastically different social institutions. The significantly higher levels of fairness among Sukuma members are attributed to two important institutional factors between the two groups: Firstly, unlike Pimbwe whose loosely linked clans are controlled by a single chief in a central village, the Sukuma (the original inhabitants of the area) live in multiple chiefdom system with a high level of cooperation among chiefdoms. Secondly, Pimbwe has smaller scale cooperative units, mostly limited to family and friends within each village, whereas in Sukuma there are larger scale cooperative units at the village level that go beyond family boundaries. The average within-village (between village) offers in the UG for the Sukuma is reported 42% (4 times) higher than the Pimbwe.

Our results are in agreement with key hypotheses of these empirical findings. The determining role of *Payoffs to Cooperation* in the Henrich’s study and the impact of the scale of cooperative units on fairness in ethnic groups in the Tanzania study can be explained by the significant role of network modularity in increasing fairness norms. That is, in a society with a stronger community structure, there are more interactions within each group as opposed to between groups, hence people are more likely to be involved in group cooperation for an economic production. The role of *Market Integration* in the first study and governance structure (single chiefdom versus multiple interacting chiefdoms) can further be supported by our result which points to the impact of network hubiness on fairness norms. Clearly, the multi-chiefdom governance of the Sukuma suggests a social structure with higher level of hubiness compared to Pimbwe. We also expect societies with high market integration to include entities with which a large number of people have social interactions (as opposed to relying on home-grown products which results in a flat structure).

Additionally, cross-organizational studies of fairness suggest structural parameters that are related to hubiness. Cross-organizational study of fairness shows that the more connections there are from employees to managers, the higher the perception to fairness is^13^. In organizations with such structure, managers play the role of the hub and the more connections to the hub results in a more skewed distribution of number of interactions of individuals. Another study that investigates fairness across organizations shows that for a given number of workers, the lower the organization complexity (defined by the number of levels of hierarchy), the higher the perception of fairness is^11^. That is, for a fixed number of individuals, the decrease in the number of organizational levels results in having few individuals with a larger number of interactions and a more skewed degree distribution in the network of social interactions.

Our model determines structural features whose variations cause changes in the collective fair behavior. However, even the most similar results from empirical studies imply that fairness co-varies with the structural changes of social network. A future direction is to experimentally evaluate the causality between structural features of social network and variations of fairness, and create setups comparing similar *societies* side by side where the only difference is the connectivity structure of individuals’ social interactions. In order to overcome common hurdles of running such experiments in physical labs (e.g., recruiting large number of subjects), one can use the online labor market as subject pool^55^. Finally, results of this work can help with future directions of empirical research in fairness norms: So far, the variations in fairness norms have been mostly observed in non-industrial societies with drastically different institutional structures. Our results can be used to design better cross-societal studies within industrial countries by targeting sub-groups whose social interactions vary according to the findings of this research.

## Methods

### Model

Since the focus of our investigation is on the effect of interaction structure, we do not model additional mechanisms such as reputation^56^, empathy^57^, and spite^58^ that have been used in previous computational UG models. We modeled the UG on graphs^31, 50, 51, 59–61^ and employed a computational agent-based framework consisting of four levels^37^: game (behavioral), strategy, evolution, and network. At the behavioral level, we use a one-shot pairwise UG^62^ and without loss of generality we assumed the amount to be divided is one unit. The strategy of each agent *i* ∈ {1, …, *N*} is characterized by *s*_*i*_ = (*p*_*i*_, *q*_*i*_), where *p*_*i*_ ∈ [0, 1] is the amount that *i* offers when she acts as the proposer and *q*_*i*_ ∈ [0, 1] is the acceptance threshold when she plays the role of the responder. In each generation, every agent *i* plays the UG with all agents *j* in her neighborhood with a fixed strategy and equal probability of being the proposer or the responder i.e., in each interaction between two given agents, one of the agents is randomly and with equal chance selected as the proposer and the other agent is assigned as the responder (in some alternative models the chance of being the proposer/responder can be a function of the agent’s degree or the outcome of its previous interactions^63^). The payoff of an agent *i* in interaction with agent *j* is calculated according to Equation 1. We considered two conditions for the game score: (1) total accumulated payoff of each agent *i* is used as the game score, ${{\rm{\Pi }}}_{i}^{T}$, (2) For each agent *i* the accumulated payoff is normalized by the agent’s degree (*k*_*i*_) in the network and is used as the game score, ${{\rm{\Pi }}}_{i}^{{k}_{i}}$ (Equation 2).

1 $${{\rm{\Pi }}}_{i\leftarrow j}=\{\begin{array}{cc}1-{p}_{i} & {\rm{if}}\,i\,{\rm{acts}}\,{\rm{as}}\,{\rm{the}}\,{\rm{proposer}}\,(j\,{\rm{acts}}\,{\rm{as}}\,{\rm{the}}\,{\rm{responder}})\,{\rm{and}}\,{p}_{i}\ge {q}_{j}\\ 0 & {\rm{if}}\,i\,{\rm{acts}}\,{\rm{as}}\,{\rm{the}}\,{\rm{proposer}}\,(j\,{\rm{acts}}\,{\rm{as}}\,{\rm{the}}\,{\rm{responder}})\,{\rm{and}}\,{p}_{i} < {q}_{j}\\ {p}_{j} & {\rm{if}}\,j\,{\rm{acts}}\,{\rm{as}}\,{\rm{the}}\,{\rm{proposer}}\,(i\,{\rm{acts}}\,{\rm{as}}\,{\rm{the}}\,{\rm{responder}})\,{\rm{and}}\,{p}_{j}\ge {q}_{i}\\ 0 & {\rm{if}}\,j\,{\rm{acts}}\,{\rm{as}}\,{\rm{the}}\,{\rm{proposer}}\,(i\,{\rm{acts}}\,{\rm{as}}\,{\rm{the}}\,{\rm{responder}})\,{\rm{and}}\,{p}_{j} < {q}_{i}\end{array}$$

2 $$\begin{array}{c}{{\rm{\Pi }}}_{i}^{T}=\sum _{j\in N(i)}{{\rm{\Pi }}}_{i\leftarrow j}\\ {{\rm{\Pi }}}_{i}^{{k}_{i}}=\frac{1}{{k}_{i}}\sum _{j\in N(i)}{{\rm{\Pi }}}_{i\leftarrow j}\end{array}$$

Agents’ strategies evolve based on a social learning rule. The implicit assumption of this model is the ability of the agents to assess and learn from their peers—as opposed to adaptive models, in which agents learn from their previous interactions^64^. The evolutionary strategy update process is as follows. Every agent *i* randomly selects an agent *j* in the neighborhood, if the score of *j* is greater than the score of *i* then *i* adopts the strategy of *j* with a probability *W*(*s*_*i*_ → *s*_*j*_). The probability of the strategy adoption is given by the Fermi rule in equation 3 in which *β* is the selection intensity.

3 $$W({s}_{i}\to {s}_{j})={[1+exp(\beta ({{\rm{\Pi }}}_{i}-{{\rm{\Pi }}}_{j}))]}^{-1}$$

In order to account for innovation in each strategy update, a small perturbation is introduced in the value of the strategy vector. That is, if agent *i* adopts the strategy of *j* then (*p*_*i*_, *q*_*i*_) = (*p*_*j*_ + *δ*_1_, *q*_*j*_ + *δ*_2_) with *δ*_1_ and *δ*_2_ being randomly and independently picked from a small interval [−*δ*, *δ*]. Once the strategy update is completed, payoffs are reset to zero for the next evolutionary round.

### Network generation

Finding a set of networks that represent the interaction structure between agents in our model is challenging as they should balance the trade-off between generating the variation of structural features and the computational efficiency. We carefully selected 26 structures by changing the parameters of network formation models to sufficiently capture variations of structural features in this study (see Supplementary Information Section 1 for details). The structures selected for this study are as follows. Four Barabási-Albert networks, four Watts-Strogatz networks, five Tree structures, four Erdős-Rényi networks, and four networks based on Watts-Strogatz model to generate required variation of average path length. The other network structures are a 2*D*-Lattice, 3*D*-Lattice, Circular Lattice, Ring, and a fully connected graph (see Supplementary Information - Figure S1). Additionally, we selected the size of the networks to keep the model computationally efficient while the effect of structure remains significant in the evolution of agent’s strategies (see Supplementary Information - Figure S3). The size of most networks used in this study is 100 nodes with two exceptions for circular lattice and 3*D* lattice which have 125 (5 × 5 × 5) nodes. We also verified that the results are not sensitive to the deviation of 100~125 nodes in the network size. We measured the skewness of degree distribution (*g*_1_) for each network structure using ${g}_{1}=\frac{{m}_{3}}{{m}_{2}^{\mathrm{3/2}}}$ where *m*_2_ and *m*_3_ are, respectively, the second and the third moments of the degree distribution. Larger values (in magnitude) of *g*_1_ indicate more skewness in the distribution^49^. To measure the strength of the community structure (*Q*), we first used the algorithm suggested in^48^ to partition the network into communities of densely connected nodes, with the nodes belonging to different communities being only sparsely connected. Next, we measured modularity index using $Q=\frac{1}{2m}\sum _{i,j}[{A}_{ij}-\frac{{k}_{i}{k}_{j}}{2m}]\delta ({c}_{i},{c}_{j})$ where, for an unweighted graph, *A*_*ij*_ represents an edge between *i* and *j*, *k*_*i*_ is the degree of vertex *i*, *c*_*i*_ is the community to which vertex *i* is assigned, function *δ*(*u*, *v*) is 1 if *u* = *v* and 0 otherwise, and *m* is the number of edges in the graph^47^.

### Numerical simulation

In each initialization, we populated the network with agents for each structure and draw a random value for *p*_*i*_ and *q*_*i*_ independently from a uniform distribution *U*(0, *H*) for each agent where *H* ∈ {0.5, 1}. Unlike similar computational models where the value at the equilibrium is calculated by averaging strategies over a certain number of generations after a transient stage with a fixed and large number of generations, we used an adaptive convergence criteria (see Supplementary Information Section 2 for details). In our model, the evolutionary process continued over a number of generations until no more than one agent updates its strategy for a consecutive window of 100 generations. To enhance convergence, we considered a noise threshold of *ε* = 0.05 for strategies below which agents do not adopt a new strategy. This approach (1) ensures that evolutionary process continues until strategies of all agents stabilize in the population and (2) is more computationally efficient as the number of generations for the convergence becomes a function of the network structure as well as the initialization. We determined the number of initializations so that the average value over all runs for each network structure is reasonably robust (see Supplementary Information Section 3 for details). We used 1024 initializations for each network structure and calculated the average value of strategies for each network. This ensures less than 1% variation in the average strategy at the equilibrium for our case. We used *β* = 0.1 for the selection intensity and *δ* = 0.005 for perturbation in strategy adoption. All runs converged for these simulation parameters across all network structures selected for this study. Although our simulation results suggest that the population becomes stable in a wide range of network structures, the analysis of the evolutionary dynamic of games on various structures is an open question^65^. Most of the models in the literature are based on the prisoner dilemma and do not consider structure^66^ or are limited to special structures^67, 68^. The analysis of the evolutionary dynamics of the UG and the stability of certain population states on networks with different structural features is an important future step of this research.

### Data Availability

The data presented in this article can be found on figshare:

https://dx.doi.org/10.6084/m9.figshare.4240031.v2

## Electronic supplementary material

Details of Model Implementation
