# Supplementary material for: Fair Topologies: Community Structures and Network Hubs Drive Emergence of Fairness Norms
Source: Sci Rep. 2017 Jun 2;7:2686. doi: 10.1038/s41598-017-01876-0 (PMC5457444; doi:10.1038/s41598-017-01876-0)
Supplement: Supplementary file 1 — Details of Model Implementation [file 41598_2017_1876_MOESM1_ESM.pdf]

Fair Topologies: Community Structures and Network Hubs Contribute to the Emergence of Fairness Norms (Supplementary Information)

Mohsen Mosleh, Babak Heydari

School of Systems and Enterprises, Stevens Institute of Technology, Hoboken, NJ 07030

Contents

1 Network Structures **2**

2 Convergence **4**

3 Robustness of Results **5**

4 Data Analysis **6**

1 Network Structures

Selecting appropriate network structure is a key challenge in our study. On the one hand, we need enough network structures that cover a wide range of topology parameters. On the other hand, the number of network structures is limited by the computational cost of simulation. In order to find the networks that generate required variations of structural features for this study, we used a wide range of deterministic (e.g., Tree, Lattice, Ring, and Full) and stochastic (e.g., Barabási-Albert (BA), Watts-Strogatz (WS), and Erdős-Rényi (ER)) network formation models. Based on these network models, we initially generated a large set of structures by changing the parameter(s) of each model. We then kept a subset of structures that sufficiently generate variations of structural features and result in different values of average strategies at the convergence.

We selected 26 network structures (Figure S1) to capture the effect of network structural features on the average strategy at the equilibrium as follows. Four BA networks (13-BA, 14-BA, 15-BA, and 16-BA in Figure S1) with $m = 3$ and $p_{pa} \in \{0.6, 1, 1.4, 1.8\}$ where m is the the number of edges that are added in each time step and p_{pa} is the power of the preferential attachment. Four WS networks (18-WS, 19-WS, 20-WS, and 21-WS in Figure S1) with $k = 4$ and $p_{rw} \in \{0, 0.2, 0.6\}$ where k is the number of nearest neighbors in the ring topology and p_{rw} is the probability of rewiring each edge. Five Tree structures (1-Tree, 2-Tree, 3-Tree, 4-Tree, and 5-Tree Figure S1) with the number of children of each vertex, $n_{chi} \in \{2, 4, 6, 8, 10\}$. Four ER networks (22-ER, 23-ER, 24-ER, and 25-ER Figure S1) with the probability for edge creation, $p_{ec} \in \{0.2, 0.4, 0.6, 0.8\}$. Four networks based on WS model (7-WS, 8-WS,9-WS, and 10-WS Figure S1) to generate required variation of average path length. The other network structures are a $2D$-Lattice (11-Lattice in Figure S1), $3D$-Lattice (12-Lattice in Figure S1), Circular Lattice (17-Lattice in Figure S1), Ring (6-Ring in Figure S1), and a fully connected graph (26-Full in Figure S1).

Figure S2 depicts how the selected network structures cover a range of variations of structural features. Variations in average path length (path) and girth are mainly generated

by the WS model. Variations of skewness of degree distribution (hubiness) are generated by the BA and the Tree network models. Skewness of degree distribution increases by the increased number of children in Tree and increased power of preferential attachment in BA. The higher values of power of prefential attachement in the BA model enhance the tendency to link to highly connected nodes and accelarate the rich-gets-richer process. This results in a hub-and-spoke structure where most nodes are connected to a few central nodes [1]. The consequence of higher power of preferential attachment for networks with fixed average degree is higher skewenewss of degree distribution since the connections are shifted from a majority of nodes to a few hubs. Variations in transitivity and average degree (degree) are generated by the ER model in which both degree and transitivity increase by increasing the probability of connection between two arbitrary nodes. Finally, variations of modularity are mainly generated by the WS model in which network modularity decreases with increased probability of rewiring. The effects of variation of community structures obtained from the WS model and those structures generated by adjusting modularity based on other methods, such as [2], on the results of evolutionary games simulation were validated in previous work [3].

To balance the trade-off between computational efficiency and sensitivity to structural variations, we did a sensitivity analysis of average strategies at equilibrium against network size for different network models' parameters. For each network model, we selected two (three) network parameters and then compared the value of strategies against network size. Figure S3 depicts the results of network size sensitivity analysis for four network models. We compared two BA networks with $m = 3$ and $p_{pa} \in \{1, 1.8\}$ (where m is the the number of edges that are added in each time step and p_{pa} is the power of the preferential attachment), three types of lattice structures (i.e., $2D$ lattice, circular lattice and $3D$ lattice), two tree structures where the number of children of each vertex is proportional to the network size (i.e., %8 and %16 of the total number of nodes), and two WS structures with $k = 4$ and $p_{rw} \in \{0, 0.4\}$ (where k is the number of nearest neighbors in the ring topology and p_{rw} is

the probability of rewiring each edge).

As depicted in Figure S3, for small network size, the difference in average strategies is not significant for different values of the network models. We selected network structures with 100 nodes (with two exceptions: circular lattice and $3D$ lattice which have 125 ($5 \times 5 \times 5$) nodes, where the results are not sensitive to the deviation of $100 \sim 125$) that capture the effect of varying parameters across all network models that are analyzed. It is worth noting that the average strategies also change with network size for certain network models and parameters values as structural features are also affected by the network size for fixed network parameters. For example, in WS model the average offer value becomes fix for larger network size when $p_{rw} = 0$, however, it decreases with the network size when $p_{rw} = 0.4$.

2 Convergence

A common practice to calculate the value at the equilibrium is to average strategies over a certain number of generations after a transient stage with fixed and large number of generations [4, 5, 6, 7]. This, however, has two main shortcomings. Firstly, it may not be computationally efficient to consider a fixed and large number of generations for the transient stage of all simulation runs as it can be different from one network structure and initialization to another. Secondly, one needs to ensure that the agents' strategies are in fact stabilized after the transient stage, otherwise the averaged strategies would be misleading.

In this study, we used the adaptive convergence criteria suggested in [3]. For each initialization, the simulation of the evolutionary process continues over a number of generations until no more more than one agent (or a certain percentage of the whole population) updates its strategy for a consecutive window of 100 generations. To enhance convergence, we considered a noise threshold of $\epsilon = 0.05$ for strategies below which agents do not adopt a new strategy. All runs converged for these parameters. As a result, the number of generations

before convergence becomes a strong function of the network structure as it is depicted in Figure S4.

Figure S4(a) shows the average number of generations before convergence versus network structures. Ring requires largest number and the BA networks with $m = 3$ and $p_{pa} \in \{0.6, 1\}$ require the least number of generations among the structures selected for this study. Figure S4(b) presents the average computation time required for one simulation run for each network structure. The simulations were run on a 256-core Cray XE6 cluster (core speed=2.5GHz and L3 cache=16MB). Full graph and ER network with high average degree have the highest and BA networks with $m = 3$ and $p_{pa} \in \{0.6, 1\}$ have the lowest average computation time. Comparing Figure S4(a) and (b) implies how the adaptive convergence criteria can significantly save computation time (e.g., on average, simulation on the full graph with largest number of interactions in each generation converges with relatively low number of generations).

3 Robustness of Results

In order to find the required number of initializations, we analyzed the difference between averaged strategies at the equilibrium versus the number of initializations for all network structures. For each number of initializations, we collected 10 samples of the average strategies at the equilibrium. Figure S5 and S6 show the results for each network structure, respectively, for offer value and acceptance threshold. The results indicate that for 1024 initializations that is selected for this study, variation of average strategy at the equilibrium is less than 0.01.

The results of our initial sensitivity analysis also shows that the average strategy at equilibrium does not change much with varying selection intensity (β), yet it affects the number of generations before convergence as its increased value expedite the strategy update. For this study, we used β=0.1.

4 Data Analysis

Based on the simulation results, we created a 2 × 11 data table (Table S1). Each row in the data table represents one network structure and contains average offer value, acceptance threshold, number of generations before convergence, computation time, and the network structural features. We then use this data table (excluding number of generations and computation time) as the input to our Principal Component Analysis (PCA).

Table S2 shows the loading of the simulation variables and network structural features on the Principal Components (PC) where average offer is highly correlated with PC1 and moderately correlated with PC2. Among the structural features, modularity is highly correlated with PC1, while degree is negatively correlated with PC1. Skewness is highly correlated with PC2. We selected the first two PCs that explain more than %80 of the total variations in the dataset according to the scree plot shown in Figure S7(a). The *biplot* [8] in Figure S7(b) shows the correlation between initial variables (i.e., average strategies and structural features) based on the first two PCs Corresponding values for each network structures are also shown on the plot based the first two PCs.

References

[1] Albert-László Barabási. Network science, 2016.

[2] Andrea Lancichinetti, Santo Fortunato, and Filippo Radicchi. Benchmark graphs for testing community detection algorithms. *Physical review E*, 78(4):046110, 2008.

[3] David A Gianetto and Babak Heydari. Network modularity is essential for evolution of cooperation under uncertainty. *Scientific reports*, 5, 2015.

[4] Te Wu, Feng Fu, Yanling Zhang, and Long Wang. Adaptive role switching promotes fairness in networked ultimatum game. *Scientific reports*, 3, 2013.

[5] Reiji Suzuki, Tomoko Okamoto, and Takaya Arita. Emergent dynamics of fairness in the spatial coevolution of proposer and responder species in the ultimatum game. *PloS one*, 10(1):e0116901, 2015.

[6] Jia Gao, Zhi Li, Te Wu, and Long Wang. The coevolutionary ultimatum game. *EPL (Europhysics Letters)*, 93(4):48003, 2011.

[7] MN Kuperman and S Risau-Gusman. The effect of the topology on the spatial ultimatum game. *The European Physical Journal B*, 62(2):233–238, 2008.

[8] Karl Ruben Gabriel. The biplot graphic display of matrices with application to principal component analysis. *Biometrika*, 58(3):453–467, 1971.

Struct.	$\tilde{q}$	$\tilde{p}$	Gen.	Time(s)	Deg.	Girth	Mod.	Path	Skew.	Trans.
1-Tree	0.21	0.34	422	6.05	1.98	0	0.76	5.1	1.17	0
2-Tree	0.21	0.36	463	6.61	1.98	0	0.81	3.94	2.3	0
3-Tree	0.21	0.37	539	7.69	1.98	0	0.8	3.5	3.06	0
4-Tree	0.23	0.4	455	6.48	1.98	0	0.78	3.36	3.67	0
5-Tree	0.26	0.42	439	6.26	1.98	0	0.76	3.23	4.13	0
6-Ring	0.24	0.33	1598	22.44	2	100	0.8	25.25	0	0
7-WS	0.25	0.34	904	12.88	2	29	0.79	12.04	0.79	0
8-WS	0.24	0.34	828	11.79	2	17	0.8	15.71	0.37	0
9-WS	0.25	0.34	1312	18.62	2	78	0.8	21.99	0	0
10-WS	0.23	0.34	604	8.64	2	13	0.79	8.86	1.2	0
11-Lattice	0.23	0.33	325	5.34	3.6	4	0.65	6.67	-1.06	0
12-Lattice	0.2	0.3	307	7.01	4.8	4	0.58	4.84	-0.24	0
13-BA	0.17	0.3	206	4.13	5.88	3	0.33	2.55	3.57	0.1
14-BA	0.19	0.32	205	4.09	5.88	3	0.28	2.31	4.3	0.11
15-BA	0.2	0.35	445	8.7	5.88	3	0.2	1.99	4.57	0.09
16-BA	0.21	0.37	990	19.18	5.88	3	0.17	1.95	5.24	0.08
17-Lattice	0.17	0.28	261	6.53	6	4	0.43	3.63	0	0
18-WS	0.24	0.33	402	8.2	8	3	0.67	6.7	0	0.64
19-WS	0.17	0.29	243	5.11	8	3	0.45	2.52	-0.03	0.19
20-WS	0.17	0.29	238	5.08	8	3	0.32	2.44	0.32	0.11
21-WS	0.17	0.29	230	4.84	8	3	0.29	2.41	0.41	0.07
22-ER	0.15	0.27	293	11.17	20.18	3	0.16	1.81	0.25	0.21
23-ER	0.15	0.26	301	18.49	38.4	3	0.1	1.61	0.14	0.39
24-ER	0.15	0.26	291	25.27	57.92	3	0.06	1.41	0.09	0.58
25-ER	0.15	0.26	334	37.97	79.4	3	0.03	1.2	-0.22	0.8
26-Full	0.15	0.26	323	43.89	99	3	0	1	0	1

Table S1: **The data table created based on simulation results.** Each row represents one network structure and shows average strategy at the equilibrium, average time and generations for converge, and the network structural features.

	**PC1**	**PC2**	**PC3**	**PC4**	**PC5**	**PC6**	**PC7**	**PC8**
$\tilde{p}$	**0.815**	**0.437**	0.332	-0.120	0.048	-0.071	-0.091	0.062
$\tilde{q}$	0.913	-0.048	0.263	-0.241	-0.152	-0.093	0.036	-0.059
Degree	**-0.837**	-0.258	0.418	-0.107	0.188	-0.102	0.044	-0.018
Girth	0.487	-0.755	0.185	0.381	0.036	0.020	-0.101	-0.036
Modularity	**0.896**	-0.100	-0.065	-0.369	0.162	0.141	0.011	-0.022
Path	0.667	-0.713	0.111	0.130	-0.021	0.007	0.110	0.062
Skewness	0.268	**0.792**	0.375	0.382	0.024	0.085	0.062	-0.020
Transivity	-0.802	-0.240	0.461	-0.229	-0.126	0.135	-0.032	0.015

Table S2: **The correlations between the initial variables and the independent principal components.** Significant correlations are in bold. Average offer is highly correlated with PC1 and moderately correlated with PC2. Modularity is highly correlated with PC1, while Degree is negatively correlated with PC1. Skewness is highly correlated with PC2.

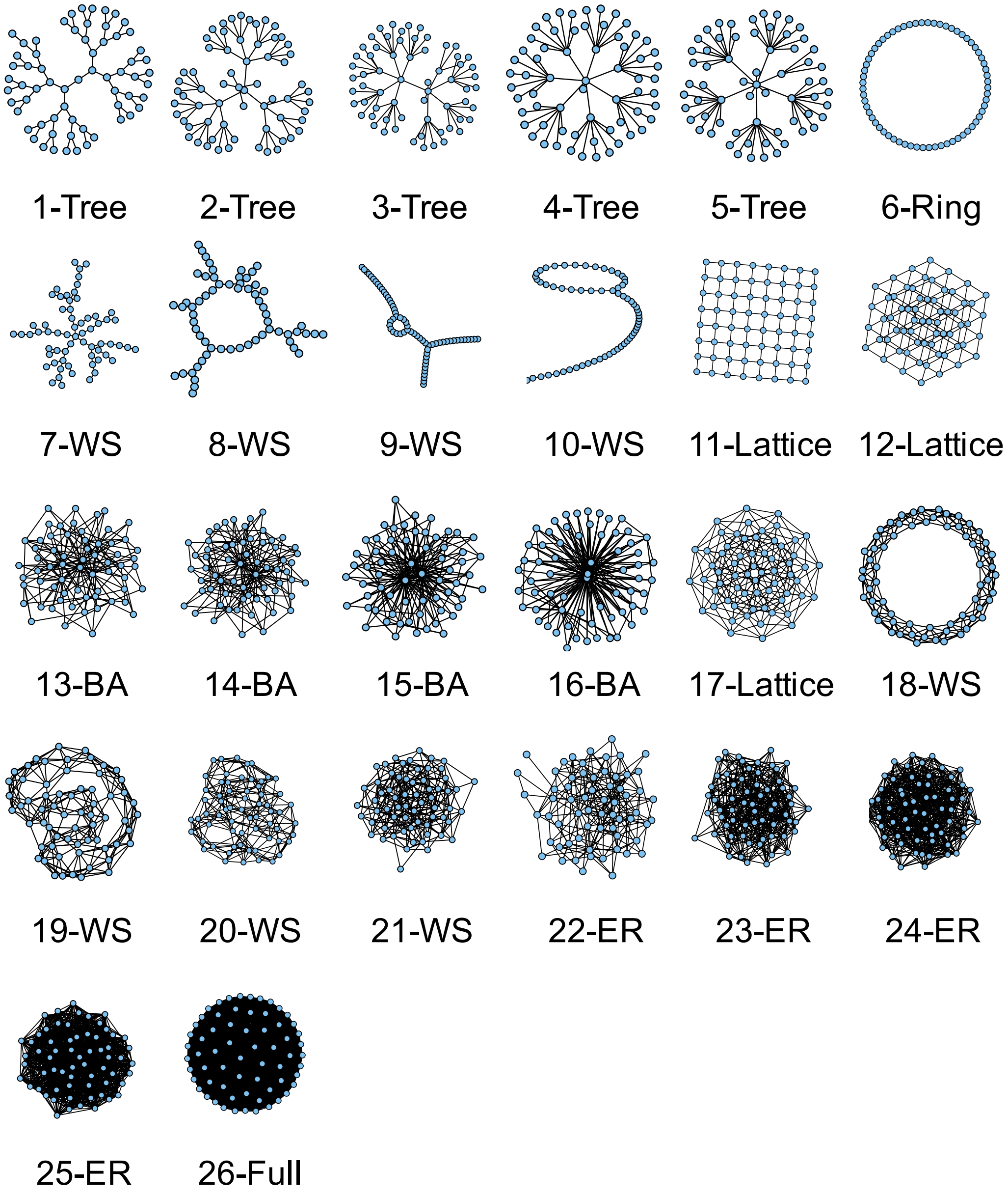

Figure S1: **Network structures selected for this study.** The network models used to generate this structures includes: Barabási-Albert (BA) Scale-Free, Watts-Strogatz (WS) Small-World, Erdős-Rényi (ER) Random graph, Lattice, Tree, and full graph. Graph parameters and number of nodes are adjusted for the visualization purpose.

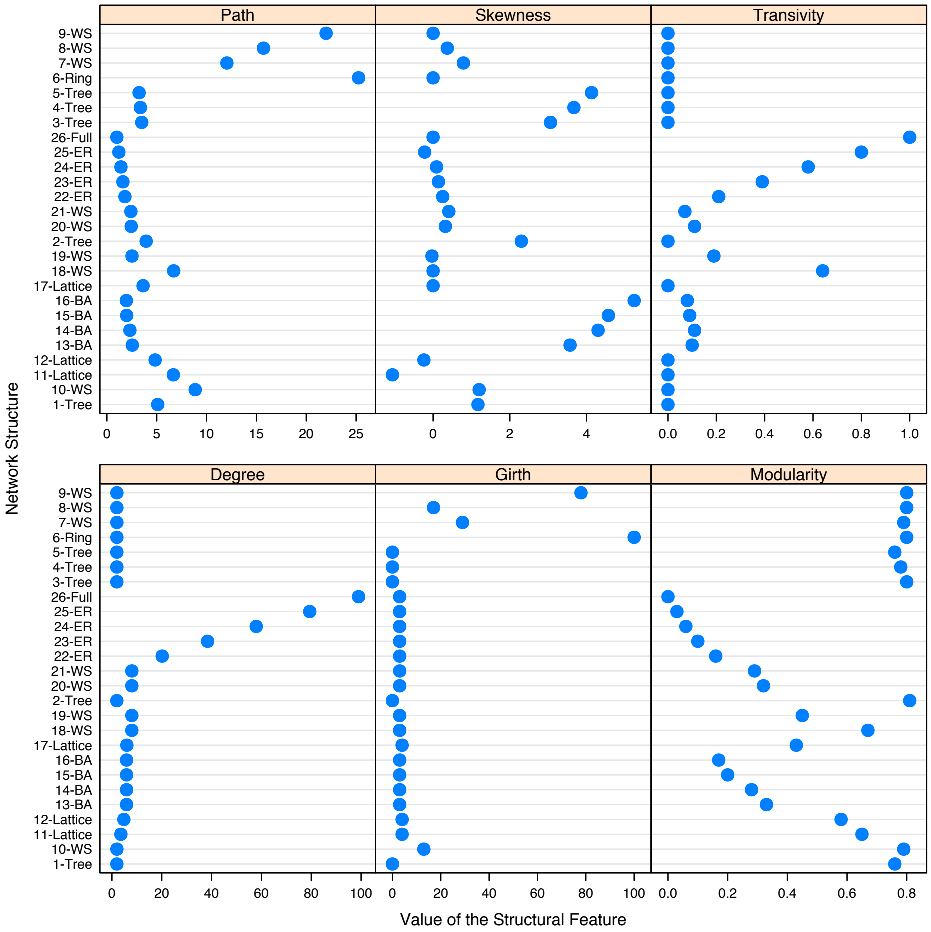

Figure S2: **Variations of structural features across different network structures selected for this study.** Each panel represents the value of one structural feature against different network structure.

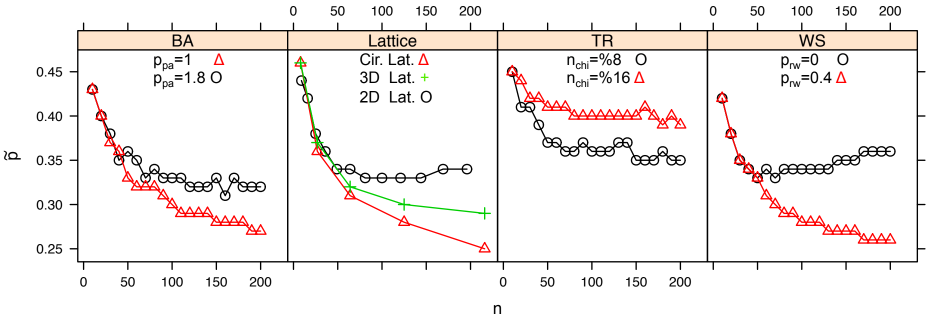

(a)

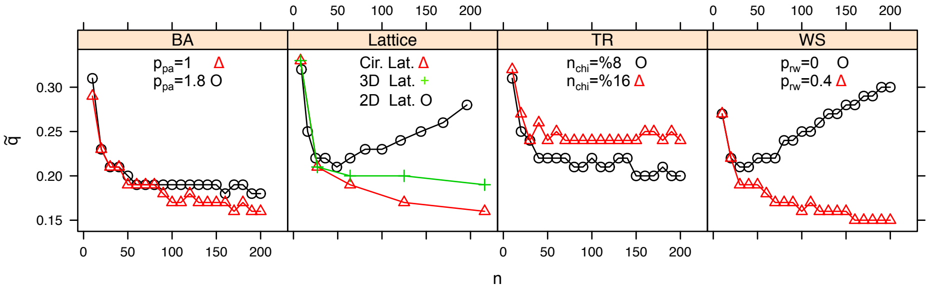

(b)

Figure S3: **Average strategy versus network size for different network models and the network models' parameters.** Each panel represents one network model and shows the value of average strategy (i.e., (a) offer value, and (b) acceptance threshold) at the equilibrium versus network size for two (three) network model parameters.

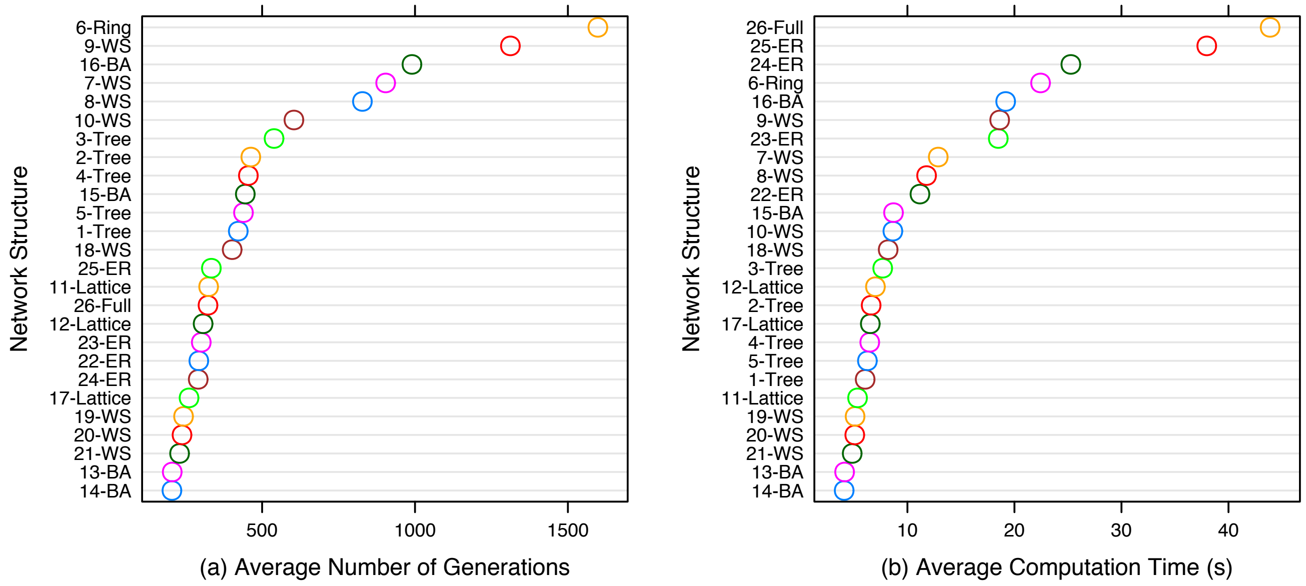

Figure S4: **(a) Average number of generations before reaching the equilibrium for each network structure, (b) Average computation time in seconds before reaching the equilibrium for each network structure.** Network structures are listed in descending order according to the number of generations and convergence time.

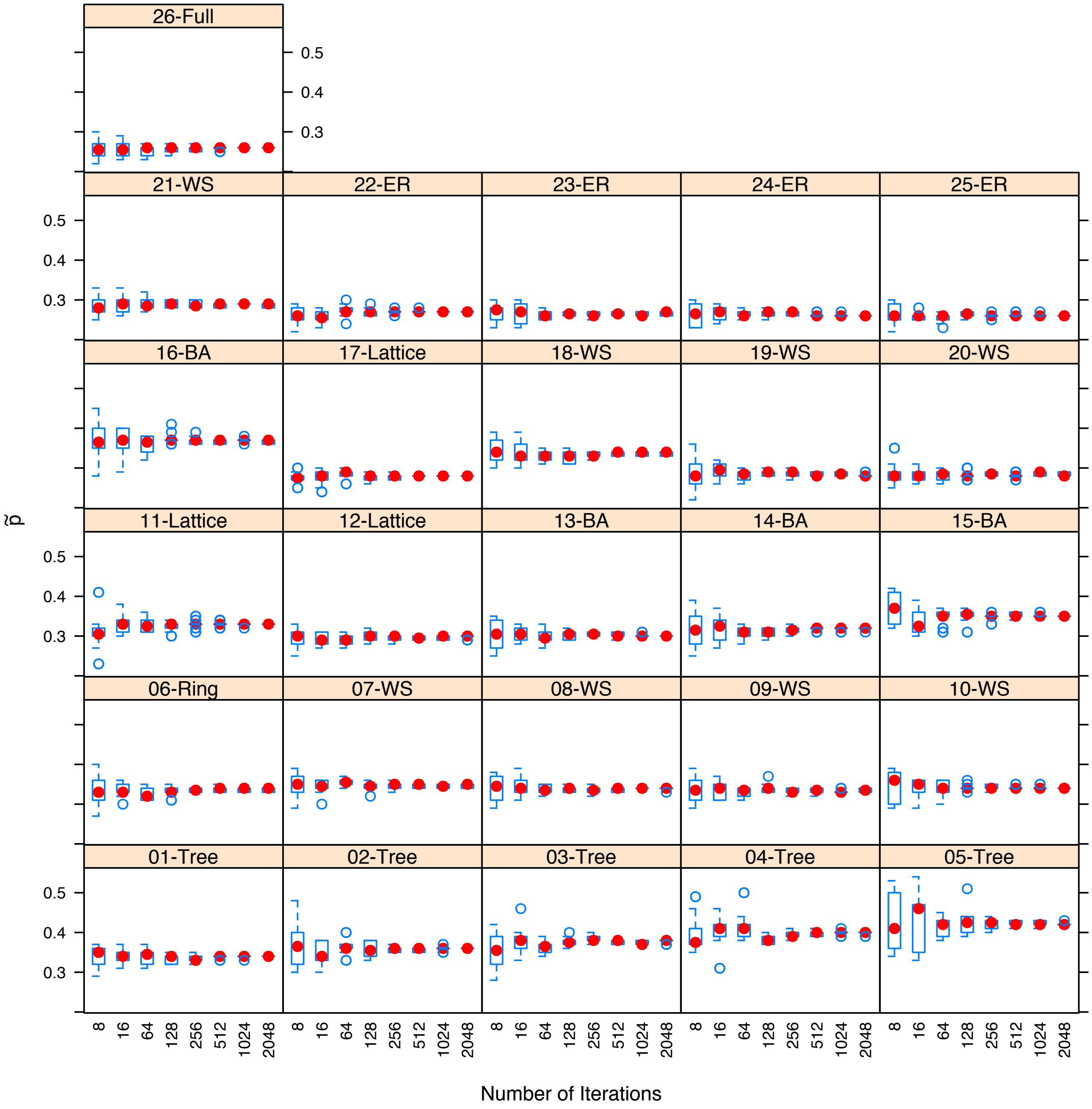

Figure S5: **Variations of averaged offer values at the equilibrium versus the number of iterations for 10 realizations.** Each panel represents the variation of average offer value for one network structure and different number of iterations.

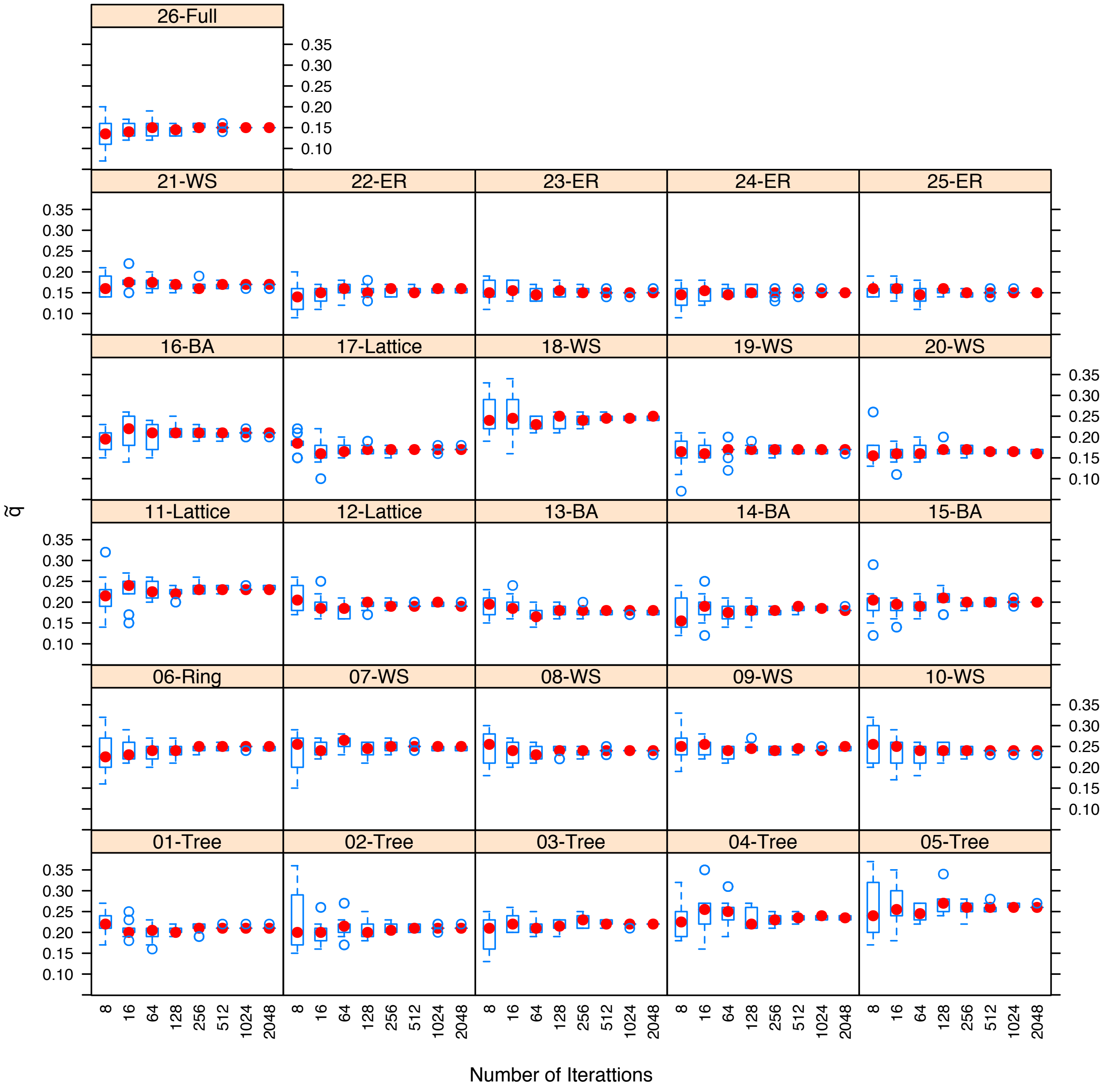

Figure S6: **Variations of the averaged values of acceptance threshold at the equilibrium versus the number of iterations for 10 realizations.** Each panel represents the variation of average acceptance threshold for one network structure and different number of iterations.

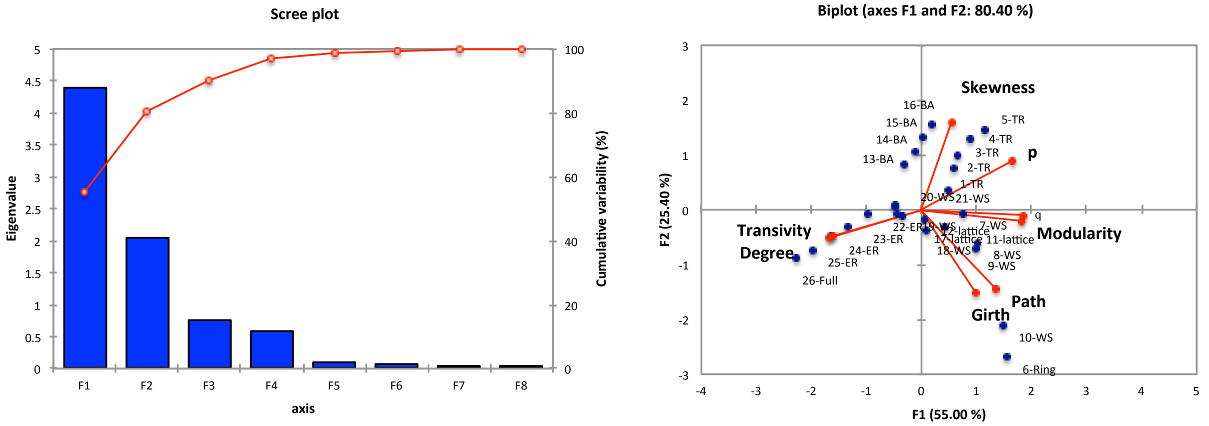

(a) (b)

Figure S7: **Principal Component Analysis results summary** (a) The scree plot displays the eigenvalues associated with each principal component in descending. The first two components explain more than 80% of variations in the whole dataset. (b) The biplot the correlation between initial variables based on the first two principal components. The angles between the variables represent the level correlation.
